# Dynamics of cell polarity in tissue morphogenesis: a comparative view from *Drosophila* and *Ciona*

**DOI:** 10.12688/f1000research.8011.1

**Published:** 2016-06-02

**Authors:** Michael T. Veeman, Jocelyn A. McDonald

**Affiliations:** 1 Division of Biology, Kansas State University, Manhattan, Kansas, 66506, USA

**Keywords:** Border cells, notochord, collective migration, morphogenesis, intercalation, cell polarity, Par polarity complex, planar cell polarity

## Abstract

Tissues in developing embryos exhibit complex and dynamic rearrangements that shape forming organs, limbs, and body axes. Directed migration, mediolateral intercalation, lumen formation, and other rearrangements influence the topology and topography of developing tissues. These collective cell behaviors are distinct phenomena but all involve the fine-grained control of cell polarity. Here we review recent findings in the dynamics of polarized cell behavior in both the *Drosophila* ovarian border cells and the *Ciona* notochord. These studies reveal the remarkable reorganization of cell polarity during organ formation and underscore conserved mechanisms of developmental cell polarity including the Par/atypical protein kinase C (aPKC) and planar cell polarity pathways. These two very different model systems demonstrate important commonalities but also key differences in how cell polarity is controlled in tissue morphogenesis. Together, these systems raise important, broader questions on how the developmental control of cell polarity contributes to morphogenesis of diverse tissues across the metazoa.

## Introduction

Cells in developing tissues exhibit numerous types of polarity, both with respect to the tissue itself and to the axes of the organ or embryo. The fundamentals of cell polarity, especially epithelial polarity, in the early embryo are relatively well understood. Great progress has been made in the decades since the discovery of the Partitioning Defective (Par) genes in *Caenorhabditis elegans* ^1, 2^ and the Planar Cell Polarity (PCP) genes in *Drosophila* ^3– 6^. Throughout animal development, however, various types of cell polarity either have to be maintained or dramatically remodeled for groups of cells and tissues to undergo large scale morphological changes. The mechanisms of how cell polarity is controlled in tissues undergoing complex movements and rearrangements remain under active investigation.

In this review, we describe the current knowledge of how cell polarity is regulated in two developmental model systems: the *Drosophila* border cells and the *Ciona* notochord. Border cells undergo a directed collective migration through an actively developing tissue, whereas the *Ciona* notochord forms through a series of intricate morphogenetic events, including mediolateral intercalation, cell shape changes, and lumen formation. The *Drosophila* border cells and *Ciona* notochord cells both undergo complex, multi-stage tissue morphogenesis processes. Although collective directional migration and mediolateral intercalation are very different, both involve the coordinated behaviors of groups of cells that exhibit multiple, distinct, highly dynamic axes of polarity. While the Par/atypical protein kinase C (aPKC) pathway and the PCP pathway are involved in both border cells and notochord, they vary considerably in their precise roles and relative importance. The seemingly disparate border cell and notochord models highlight important concepts in how different kinds of cell polarity contribute to developing organs and tissues, at both small and large scales.

## Cell polarity in the *Drosophila* ovarian border cells

**Figure 1.**
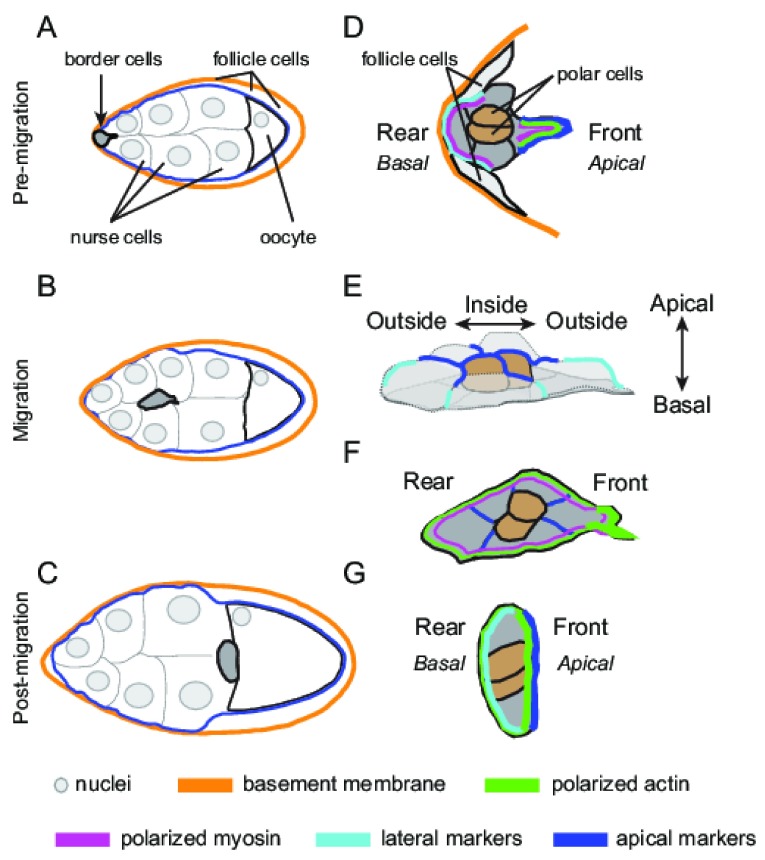
Multiple developmental polarities in *Drosophila* border cell migration. ( **A**– **C**) Schematic of egg chambers showing the stages of border cell migration during ovarian development. Border cells form at the anterior end of the egg chamber ( **A**), migrate between nurse cells ( **B**), and reach the oocyte at the posterior end ( **C**). For simplicity, individual follicle cell membranes are not shown. ( **D**– **G**) Close-up view of border cell clusters, and the variety of cell polarities displayed by border cells, at the indicated stages of migration. Polar cells (brown) are always at the center of the cluster. The morphological cell polarities correspond to polarized actin, myosin, lateral, and apical markers, as shown in the key. ( **D**) Pre-migration stage. Border cells exhibit a clear front-rear polarity. Prior to the movement between nurse cells, border cells detach from the basement membrane and delaminate from adjacent epithelial follicle cells. ( **E**, **F**) Migration stage. Two views of the same cluster are shown: a three-dimensional view ( **E**) and a two-dimensional view through the middle of the cluster ( **F**). At this stage, border cells display inside-outside ( **E**), apical-basal ( **E**) and front-rear ( **F**) polarities. ( **G**) Post-migration stage. Once border cells reach the oocyte, they orient with the apical side touching the oocyte.

Many cell types undergo coordinated multicellular migration in embryogenesis. These so-called migrating “collectives” need to polarize at the group level so that they can reach the correct place at the right time and populate (or produce) tissues and organs with the appropriate orientation. The *Drosophila* ovarian border cells provide a simple genetic system in which to understand the mechanisms that control collective migration ( Figure 1A–C). The *Drosophila* ovary consists of multiple strings of progressively more mature egg chambers, each of which produces a fertilized embryo ^7^. The egg chamber consists of the oocyte and 15 supporting nurse cells in the center, surrounded by a monolayer of polarized epithelial follicle cells ( Figure 1A). In mid-oogenesis, between four and eight follicle cells at the anterior end are induced to form a cluster by a specialized pair of cells called the polar cells. The border cell cluster (including the polar cells) then delaminates from the epithelium. Border cells migrate as a group while navigating their way between the nurse cells to the anterior border of the oocyte, where they stop. The border cell cluster contributes to the formation of the micropyle, which is the sperm-entry pore in the eggshell and is required for fertilization of the oocyte ^8^.

Border cells exhibit and require multiple forms of cell polarity. Border cells initially display a canonical apical-basal polarity because they delaminate from an existing epithelium. For both the follicle cells and the presumptive border cells, the apical side of each cell faces the inside of the egg chamber, contacting the nurse cells and oocyte ( Figure 1A). The basal side, on the outer edge of the egg chamber, contacts the basement membrane. The apical side of all border cells thus initially points towards the oocyte and is enriched for the apical complex of Par/aPKC cell polarity proteins: aPKC, Par-3 (called Bazooka, or Baz, in flies), and Par-6 ^9, 10^. The apical edge (front) produces F-actin- and non-muscle myosin II- (myosin-) enriched migratory protrusions ^11– 13^. The basolateral polarity proteins Par-1 and Discs large (Dlg) are found at the back, or rear, of the cluster ( Figure 1D) ^14^. Visible membrane extensions at the back must retract for border cells to move away from the epithelium.

As soon as border cells move into the egg chamber, however, they undergo a poorly understood ~90° rotation. The apical side now reorients orthogonal to the direction of migration, as visualized by a strong enrichment of Par-6 and Par-3 proteins at the “top” of the cluster ( Figure 1E) ^9^. In a cross-sectional view through the cluster, the polar cells are positioned in the middle, surrounded by border cells radiating out in a “rosette” pattern ( Figure 1F) ^15^. The apico-lateral contacts between border cells are enriched for both the Par/aPKC complex proteins and the apical Crumbs/Stardust/PatJ complex ^9, 15, 16^. The cell adhesion proteins E-cadherin and integrin are located in a complementary pattern along internal basolateral membrane contacts between border cells ^15, 17^. Par-3 and Par-6 are required for the characteristic rosette conformation of the cluster during their migration, as well as organization of these other membrane-enriched proteins ^9, 17^. At this stage, protrusions now extend from basolateral cell membranes ( Figure 1E). As border cells finish their migration, they undergo another rotation, turning so that their apical side again faces the oocyte ( Figure 1C,G). This orientation may help establish a continuous epithelial layer that ultimately encloses the oocyte ^9^. Border cells thus retain epithelial apical-basal cell polarity, but this polarity is complex and varies during the course of migration.

A second type of polarity exhibited by border cells is front-rear polarity ( Figure 1D,F,G). All migrating cells produce major cellular protrusions at the front that range from thin filopodia to broad lamellipodia ^18^. Front-directed protrusions help cells adhere to migratory substrates, sense directional signals from the environment, and propel the cell forward. Adhesions need to be disassembled in order to retract the cell membrane at the rear ^19^. Directionally migrating collectives produce a characteristic “super-cellular” front to rear orientation across a large number of cells ^20^. In response to a chemotactic guidance signal, border cells extend one or two prominent actin-rich protrusions from the front cell, but not from other cells ( Figure 1D–F) ^21^. Multiple growth factors secreted from the oocyte activate receptor tyrosine kinases (RTKs) on border cells ^22– 24^. A well-characterized RTK signaling pathway then stimulates the formation of a protrusion, thus setting up front-rear polarity of the border cell group ^21, 25– 28^.

Finally, border cells exhibit “inside-outside” polarity ( Figure 1E) ^29^. The free edge of the cluster establishes external contacts with the migratory substrate, the nurse cells. The rear of each border cell contacts a polar cell at the cluster center, thus producing an inner edge. Inside-outside polarity is reinforced as each border cell stays attached to neighboring cells during their journey. Such orientation appears to prevent migratory protrusions from forming internally between cells of the cluster, similar to other collectives ^29– 31^. Adhesion of border cells to the polar cells by E-cadherin is critical for establishing this polarity ^28^.

## Cell polarity in the *Ciona* notochord

**Figure 2.**
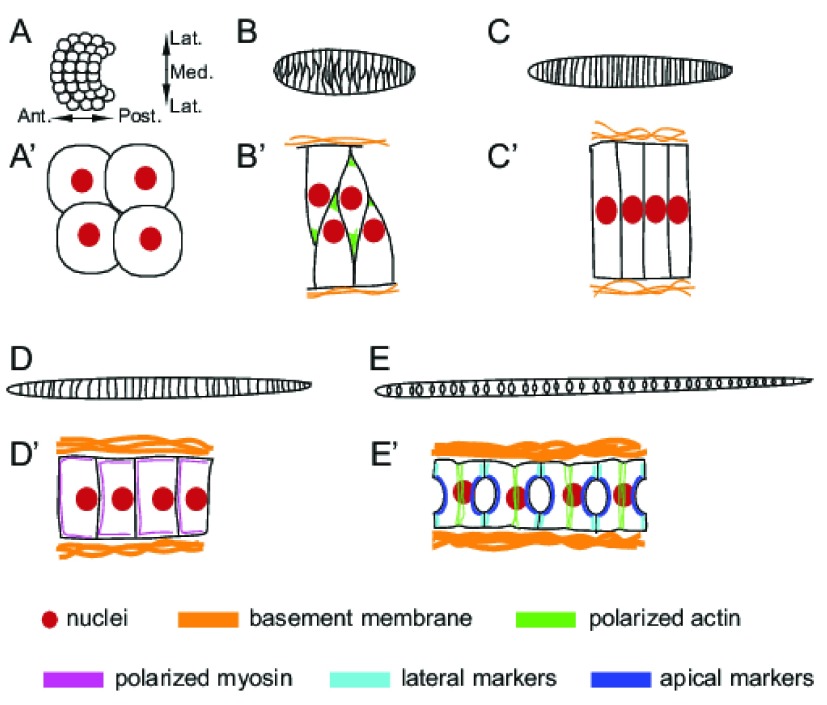
Cell polarity in *Ciona* notochord morphogenesis. ( **A**– **E**) Schematics of the entire notochord primordium at successive stages of notochord morphogenesis. Dorsal view with anterior to the left in all cases. ( **A**’– **E**’) Zoomed in views showing just a few notochord cells with greater detail. Cell nuclei, basement membranes, polarized actin and myosin, and polarized apical and lateral markers are shown as in the key at the bottom of the figure. ( **A**, **A**’) The notochord initially forms a flat plate of cells that is relatively isodiametric in the AP (anterior-posterior) and ML (mediolateral) axes. No AP or ML polarity has been described at this stage, but there is a slight asymmetry of apical-basal markers on the ventral and dorsal sides (not seen in this dorsal view). ( **B**, **B**’) The notochord cells become mediolaterally elongated and aligned as they begin to intercalate. The notochord as a whole extends along the AP axis while narrowing across the mediolateral axis. The notochord cells are clearly polarized, with a flat edge contacting the flanking tissues and actin-rich protrusions reaching across to the other side. Cell nuclei are polarized medially. ( **C**, **C**’) At the end of intercalation, the notochord forms a single-file rod of thin, disk-shaped cells (rectangular in the section shown). A perinotochordal ECM surrounds the notochord. Nuclei are located centrally and squished between the anterior and posterior cell faces. ( **D**, **D**’) The notochord cells become progressively taller in the AP dimension while narrowing mediolaterally. A new phase of AP polarity becomes evident with nuclei localized to the posterior and myosin enriched to the anterior of each cell. ( **E**, **E**’) Late in notochord morphogenesis, a distinct circumferential belt of cortical actin forms at the equator of each cell. Extracellular lumen pockets form between each notochord cell. Apical and lateral markers become distinctly polarized between the lumen pocket domain and the remaining donut shaped ring of notochord-notochord cell contacts.

The *Ciona* notochord consists of only 40 post-mitotic cells that converge and extend through mediolateral intercalation to form a single file rod ( Figure 2A–C). As seen throughout the chordates ^32^, this convergence and extension of the axial mesoderm depends on polarized motility orchestrated by the PCP pathway ^33^. Soon after their final cell division in mid gastrulation, the notochord cells become mediolaterally elongated and aligned with one another, exhibiting mediolaterally biased cell protrusions and mediolaterally biased neighbor exchanges that drive the anterior/posterior (AP) lengthening and mediolateral narrowing of the tissue ( Figure 2B,B’). It is important to emphasize that mediolateral intercalation is not a directed migration towards the embryonic midline, but instead an oriented bias in neighbor exchanges with individual cells more likely to move between their medial or lateral neighbors than their anterior or posterior neighbors.

This mediolateral polarity of tractive cell protrusions is not, however, the only manifestation of planar polarity in the *Ciona* notochord. As the notochord is intercalating it is also building a perinotochordal extracellular matrix, and the PCP pathway plays a poorly understood role in restricting laminin extracellular matrix (ECM) formation to the perinotochordal surfaces. In embryos carrying a mutation in the PCP pathway component Prickle (Pk), ectopic laminin can be seen between adjacent notochord cells and not just on the outer surfaces of the notochord ^34^. This phenotype is only seen at later stages, indicating that the PCP pathway is involved in the maintenance as opposed to the initial establishment of polarized laminin localization. It is not clear if this control is at the level of laminin secretion, assembly, stability, or otherwise, but a similar relationship between PCP signaling and polarized ECM formation has also been described in *Xenopus* ^35^.

After intercalation, the *Ciona* notochord cells are in the shape of thin, flat disks (a so-called “stack of coins”; Figure 2C,C’) but they subsequently change shape, becoming narrower across the mediolateral axis and longer along the AP axis ( Figure 2D,D’). This change from disk-shaped to drum-shaped accounts for much of the elongation of the tail and is driven by actomyosin-based contractility ^36, 37^. Several distinct aspects of cell polarity have been described during this phase of morphogenesis. Cell nuclei become localized to the posterior cell cortex, and this polarity is PCP dependent ^33^. The PCP component Pk becomes polarized to the anterior cell cortex ^33^. Myosin light chain also becomes localized to the anterior cortex in a poorly understood manner that is partially dependent and partially independent of the PCP pathway ^37^. Late in the process, a distinctive circumferential belt of actin becomes evident at the AP equator of the cell ^36^ ( Figure 2E,E’), and a recent study shows that the equatorial positioning of this belt involves a balanced interplay between PCP signaling moving the belt towards the anterior and contractility-dependent cortical flows moving it towards the posterior ^38^. The morphogenetic significance of these polarized phenomena is unclear, but it is clear that the intercalated notochord has an AP polarity that is distinct from the mediolaterally oriented planar polarity seen during intercalation.

Later in development, the *Ciona* notochord forms an inflated lumen, important for its mechanical role acting in compression to cause the larval tail to deflect from side to side instead of collapsing in response to the sequential contraction and relaxation of the left and right tail muscle cells. This central lumen originates from extracellular pockets of material formed between adjacent intercalated notochord cells ^39^ ( Figure 2E,E’). These pockets expand and then the notochord cells deintercalate somewhat to allow the pockets to connect and form a continuous lumen running the length of the notochord. This is a topologically confusing rearrangement in 3D that is difficult to depict in 2D. However, the key points are that the lumen pockets are always extracellular and the eventual fusion of multiple pockets results from altered cell-cell contacts, not from membrane fusion events. As such, this does not lead to individual notochord cells becoming donut-shaped.

This lumen formation process has been extensively studied and involves dramatic changes in cell polarity ^40– 42^. The extracellular lumen pockets form only on the anterior and posterior surfaces between adjacent notochord cells and not on the circumferential surfaces between notochord cells and the surrounding muscle, neural tube, and endodermal strand cells. The earliest signs of this polarity come from the localization of the apical markers Par3, Par6 and aPKC to the center of both the anterior and posterior cell surfaces ^41^ ( Figure 2E,E’). The basolateral markers DLG, LGL, and Scribble are excluded from this region and are found more peripherally on the anterior and posterior surfaces, as well as the circumferential surfaces that contact the perinotochordal basement membrane. Tight junctions form between these apical and basolateral domains and the extracellular lumen pockets are secreted from the apical region. This leads to the striking implication that the *Ciona* notochord forms a linear stack of cells that each possesses two distinct apical sides!

## Dynamics of developmental cell polarity: not always epithelial to mesenchymal transitions 

Cell biologists tend to think of tissues as fitting into the binary categories of epithelium versus mesenchyme. In developing embryos, however, it is clear that these distinctions are somewhat more fluid. There are many morphogenetic processes that involve distinct epithelial to mesenchymal transitions (EMT) or the reverse, mesenchymal to epithelial transitions (MET), but there are also cases where dynamic tissues exhibit both epithelial and mesenchymal characteristics. During classical EMT events, cells lose contact with each other by downregulating apical-basal polarity and E-cadherin-dependent cell-cell adhesion ^43^. Cells then detach from epithelia and the basement membrane to move through tissues. Mesenchymal cells are characterized by the ability to migrate, typically as single cells, and tend to have more elongated “fibroblast-like” shapes ^44, 45^. Coincident with becoming mesenchymal, cells often make a switch from E-cadherin to N-cadherin expression ^43, 45^. A classic example of a developmental EMT is the departure of the neural crest cells from the neural tube ^46^. Both the *Drosophila* border cells and the *Ciona* notochord, however, defy the simple characterization of epithelium versus mesenchyme.

*Drosophila* border cells, for example, display stretched shapes and extend migratory protrusions, suggesting that they are partially mesenchymal. Similar to cells that undergo EMT, border cells separate from a basement membrane and dissolve contacts with neighboring epithelial follicle cells ( Figure 1D) ^14, 21, 29^. However, border cells never lose contact with neighboring border cells or the pair of polar cells at the center of the cluster. Thus, border cells delaminate and move as a small “epithelial patch” ^15^ ( Figure 1E,F). In stark contrast to cells undergoing EMT, apical-basal polarity proteins remain localized to cell contacts between border cells ^9, 17^. Moreover, border cells upregulate E-cadherin expression and do not express N-cadherin ^15, 29^. Further emphasizing that border cells do not easily fit a stringent definition of either epithelium or mesenchyme, E-cadherin promotes the ability of border cells to migrate upon nurse cells ^15, 28^. Loss of E-cadherin in either border cells or nurse cells stops the forward movement of border cells. Dynamic and transient E-cadherin at membrane contacts between border cells and nurse cells therefore provides optimal traction for the cluster.

*Ciona* notochord cells also exhibit aspects of both epithelial and mesenchymal organization. The early notochord primordium forms a flat plate of cells with its dorsal side contacting the neural plate and its ventral side facing the archenteron. These two tissue surfaces differ in both the onset and progression of cell shape changes ^47^ and show accumulation of the apical marker aPKC ventrally and the basal marker laminin on the dorsal side ^48^. Despite these clear signs of epithelial organization, cells in this tissue are nonetheless able to undergo repeated intercalation events and change nearly all of their neighbor-neighbor relationships. After intercalation, it is not until quite late in tail extension that they again show clear signs of epithelial polarity, as they adopt their unusual biapical organization in preparation for lumen formation ^41^.

## Differing requirements for the PCP pathway

Border cells essentially move in a planar direction because the apical side relocates to the top of the cluster during migration. Three PCP pathway members, Frizzled (Fz), Dishevelled (Dsh), and Strabismus (Stbm; also known as Van Gogh), are each highly expressed in the central polar cells prior to migration, in addition to low levels in border cells ^49^. Once the cluster moves into the egg chamber, Fz localizes to the leading edge in association with F-actin rich protrusions, whereas Dsh and Stbm are found throughout the cluster ^49^. Given the PCP expression in border cells, it is surprising that loss of any one of these genes only mildly perturbs border cell migration. Knock down of *fz*, *dsh*, or *stbm* in border cells causes slight delay in cluster migration, although most clusters make it to the oocyte by the correct stage of development ^49^. Mosaic clonal analyses with PCP mutants indicate that the PCP genes help establish or maintain a lead cell identity, as well as communication between polar cells and border cells ^49^. In addition, F-actin enrichment in border cells via the RhoA GTPase pathway requires the function of the PCP genes ^49^. PCP signaling may therefore contribute both to inside-outside polarity and to front-rear polarity during border cell migration. The role for PCP pathway components is minor, however, suggesting functional redundancy with other mechanisms.

This modest role for PCP signaling in border cell migration stands in stark contrast to the diverse and essential roles of the PCP pathway in *Ciona* notochord morphogenesis. A mutation in the PCP pathway component Pk eliminates the mediolateral bias in the orientation of tractive cell protrusions and prevents the notochord plate from intercalating into a single file rod of cells ^33^. These Pk mutant embryos also show defects in perinotochordal/intranotochordal polarity, as seen by the ectopic localization of laminin to some surfaces between adjacent notochord cells ^34^. Despite the major defect in convergent extension, some cells in the posterior notochord do typically intercalate but exhibit a later defect in AP polarity, as shown by nuclei that fail to be positioned to the posterior of each cell ^33^. While it is not clear to what degree these three different aspects of cell polarity depend on one another, it is clear that the PCP pathway plays major roles in mediolateral polarity, inside versus outside polarity and AP polarity in the *Ciona* notochord.

The role of the PCP pathway in the convergence and extension of chordate axial mesoderm appears to be very broadly conserved ^32^. Interestingly, this is true even in species that employ very different cellular mechanisms of convergence and extension. In zebrafish, for example, convergence and extension is dominated by directional flows of independently migrating cells as opposed to mediolateral intercalation *per se*, but the PCP/non-canonical Wnt pathway is nonetheless essential for these movements (reviewed by 50).

Despite this conserved role in axial elongation in chordates, it is also interesting to note that the PCP pathway does not appear to be a main driver of the corresponding movement of germband elongation in the *Drosophila* embryo, which is also a tissue rearrangement defined by mediolaterally biased cell-cell neighbor exchanges ^51– 53^. A potential explanation for this discrepancy lies in the arguably distinct cellular mechanisms of intercalation. *Drosophila* germband elongation is thought to depend largely on neighbor exchanges driven by polarized contractility of mediolaterally oriented cell-cell contacts ^54, 55^, whereas research into chordate mediolateral intercalation has emphasized the role of mediolaterally polarized cell protrusion interdigitating between adjacent cells. However, recent research has shown an important potential role for polarized contractility in *Xenopus* mediolateral intercalation ^56^, and it is possible that these two modes of intercalation may be more alike than previously acknowledged, despite the differing importance of PCP signaling.

## Apical-basal polarity and morphogenesis

The apical epithelial Par/aPKC pathway plays important roles in border cell cluster organization and migration. Loss of Par-3 or Par-6, or an upstream activator c-Jun N-terminal kinase (JNK), disrupts the organization of membrane-enriched proteins, such as E-cadherin and integrin, between border cells ^9, 17^. Individual mutant border cells then partially pull away from the main body of the cluster and extend ectopic stable protrusions. Border cell clusters mutant for Par-3, Par-6 or JNK also do not complete their migration to the oocyte ^9, 14, 17^. In many directionally migrating cells, apical polarity complexes, including Par/aPKC, promote formation of the leading edge ^57– 59^. Although front-directed protrusions are critical for border cell migration, the role for Par/aPKC was unclear. A recent study connects polarized protrusion formation with apical-basal polarity at the cluster level ^16^. RTK-dependent guidance signaling triggers F-actin-rich protrusions at the cluster front through activation of Rac GTPase ^26^. Loss of Pak3 (p21-activated kinase 3) causes border cells to extend ectopic unstable protrusions and stalls their migration ^16^. Importantly, Pak3 functions downstream of the RTKs, but upstream of JNK, to control the proper localization of apical-basal polarity proteins within the cluster ^16^. The Par/aPKC apical complex thus promotes cohesion of the border cell collective and facilitates directional migration, likely through Pak3-dependent polarized protrusion extension.

The basolateral protein Par-1 has a slightly different role in border cells than the Par/aPKC apical complex. Par-1 promotes the complete separation of border cells from the adjacent follicle cell epithelium ^13, 14^. In epithelia, Par-1 at the basolateral side phosphorylates several conserved Ser/Thr residues, which prevents apical Par-3 from relocalizing to the basolateral membrane ^60, 61^. Similarly, Par-1 restricts localization of apical Par-3 at membrane contacts between border cells and adjacent follicle cells, leading to downregulation of E-cadherin between the two cell types ^14^. The mechanism by which loss of Par-3 leads to loss of cell-cell adhesion is still unclear. Polarized actomyosin contraction of the cluster rear also contributes to the delamination process ^13^. Par-1 promotes localized myosin activity to the rear of the cluster prior to its movement out of the anterior epithelium. Par-1 also plays a poorly understood role in promoting the formation of front-directed protrusions, which is independent of its role in regulating Par-3 localization ^14^. Par-1 thus contributes to front-rear polarity, at least during delamination of border cells.

While many details remain to be resolved, it is clear that canonical apical/basal polarity pathways play key roles in several aspects of border cell migration. This is quite different than in the *Ciona* notochord, where a major role for the Par/aPKC pathway has only been demonstrated quite late in tail elongation as the intercalated and elongated cells develop their unique biapical configuration in preparation for lumen formation ^41^. A modest dorsoventral asymmetry in aPKC has been described prior to intercalation in the notochord plate, but a strong asymmetry in Par/aPKC components has not been observed during intercalation or in the earlier stages of the post-intercalation notochord cell shape changes. Similarly, perturbations of the Par/aPKC pathway have been shown to have lumen secretion phenotypes but do not affect these earlier processes ^41^. It is possible that the Par/aPKC pathway has roles in intercalation that remain to be discovered, but it does not appear to be a major driver. One possible interpretation is that the intercalating notochord cells have a basal, ECM-facing, perinotochordal side but that the intranotochordal surfaces are not yet apical in any meaningful sense, instead representing a naïve condition.

A potential explanation for the very different roles of the Par/aPKC pathway in border cell migration versus notochord morphogenesis is that the border cells delaminate from an indisputably epithelial cell layer, whereas notochord morphogenesis occurs in the early embryo as the first basement membranes and the first axes of cell polarity are being established. It is clear that there are major “chicken and egg” questions about cause and effect with respect to the origins of polarized ECMs and polarized cell morphologies in early embryos.

## Knowing where you are: inside-outside polarity

The free edges of migrating collectives make contacts with the ECM of other cells. Cells at the periphery of a group thus exhibit a distinct inside-outside polarity that internal cells do not. For border cells, the internal edge contacts a central polar cell, whereas the external free edge contacts a nurse cell. The adherens junction protein E-cadherin plays a pivotal role in defining this polarity. E-cadherin is highly enriched at intra-cluster contacts between border cells and polar cells ^9, 15^. Loss of E-cadherin only in polar cells causes border cells to completely detach from the cluster ^28^. In contrast, depletion of E-cadherin only in border cells does not disrupt their adhesion to each other ^15, 28^. Thus, by keeping border cells in contact with the polar cells, E-cadherin establishes an inside-out polarity for each border cell. At the outer edge of the cluster, low levels of E-cadherin facilitate dynamic adhesion of border cells to the nurse cells ^15, 28^.

Actomyosin enrichment further defines the outer edge of the cluster. F-actin and activated myosin are normally restricted to the external edge of the cluster, with low levels found at internal border cell membranes ( Figure 1F) ^13, 27, 30^. Other migrating collectives display similarly polarized myosin at outer edges ^62– 64^. Increased activation of myosin at internal cell-cell contacts disrupts cohesion of collectives ^63^. Such polarized actomyosin activity mechanically joins cells together to promote group movement ^64, 65^. In border cells, the Hippo pathway specifically regulates the functions of actin-regulatory proteins to promote F-actin polymerization at the outer edge ^30^. Hippo prevents ectopic enrichment of F-actin and activated myosin at contacts between border cells. Actomyosin tension on the outer edge of the cluster is important for cluster motility ^27, 30^.

*Ciona* notochord cells also exhibit an inside-out polarity, with the perinotochordal basement membrane only forming on the outer surfaces touching the flanking muscle, neural tube, and endodermal strand cells, and not on the inner surfaces touching other notochord cells. This polarity is at least partially PCP dependent ^34^, but is distinguishable from the polarity that drives intercalation. In this case, perinotochordal basement membrane forms on all of the notochord-to-not notochord cell surfaces, but tractive protrusions on the notochord-to-notochord cell surfaces are heavily mediolaterally-biased ^33, 47^.

As intercalation proceeds, a distinctive ventral groove forms along the midline of the notochord primordium ^47^. It is not clear if this represents a dorsoventral difference in the speed of intercalation or a contraction of ventral cell surfaces, but it arguably transforms the dorsal surface of the notochord plate into the outer surface of the intercalated notochord. Overexpression of a dominant negative ephrin disrupts the apicobasal polarity of the notochord plate and blocks intercalation, suggesting important but cryptic functional relationships between these apicobasal, inside-out and mediolateral manifestations of polarity ^48^.

## Cytoskeletal readouts of polarity

Dynamic changes in cell polarity are ultimately manifested as polarized cell behaviors, many of which are driven by the cytoskeleton. A key distinction here is in the ability of the actomyosin cytoskeleton to both pull and push depending on the local architecture of the actomyosin network ^66^. While not the focus of this review, many important invagination and folding events in developing embryos are driven by apical constriction, in which polarized contractility plays the central role and polarized protrusions are not involved ^67^. More frequently, however, the same cell will demonstrate both protrusive and contractile behaviors. This is particularly true for migrating cells or cell collectives in which the leading edge has polarized lamellipodia and filopodia, whereas localized contractility ensures the retraction of the trailing edge. The interplay between protrusive and contractile behaviors is difficult to study because the relevant structures are small and extremely dynamic in time and space.

The convergence and extension of the *Ciona* notochord involves at least two distinct mechanisms influencing protrusive cell behaviors. The PCP pathway plays the major role in restricting the cell protrusions driving intercalation to medial and lateral surfaces ^33^, though it remains unclear what sort of broader cues are used to align this planar polarity with the axes of the embryo. As cell protrusions reach across to contact the flanking cells on the opposite side of the embryo, these protrusive behaviors quickly cease and the cell flattens out against the adjacent muscle, neural, or endodermal strand cell. This boundary capture requires an intact perinotochordal ECM, as it is badly perturbed in a mutant for a notochord-expressed laminin alpha3/4/5 ^34^. In this mutant, the notochord cells exhibit persistent motility and intercalate inappropriately between many of the cells surrounding the notochord.

The subsequent shape changes in the notochord are driven by actomyosin contractility, but it remains unclear to what extent polarized contractility is important. Myosin becomes enriched to the anterior early in this process ^37^, and a distinctive circumferential belt of actin forms somewhat later ^36^; however, it is experimentally difficult to disentangle the relative importance of contractility on these different cell surfaces.

In *Drosophila* border cells, the formation of an F-actin-rich protrusion in the lead or front cell is a major manifestation of cluster-wide front-rear polarity ^11, 12^. Regulation of polarized border cell protrusions was a mystery prior to the discovery of *ex vivo* culture conditions that supported egg chamber development ^21, 25^. Live time-lapse imaging of migrating border cells reveals the remarkable dynamics of these protrusions. Border cells can extend and retract a long lamellipodia-like protrusion multiple times before moving out of the epithelium. Retraction of this protrusion requires actomyosin contraction ^11^. Once the cluster moves into the egg chamber, only the front border cell produces a stable protrusion. Major protrusions are actively suppressed in the rest of the border cells.

A strikingly complex RTK pathway sets up which border cell will produce a migratory protrusion. Border cells are guided to the oocyte by RTK signaling ^22– 24^. Multiple growth factors are secreted by the oocyte to activate two RTK receptors, platelet-derived growth factor (PDGF) and vascular endothelial growth factor (VEGF) related receptor (PVR) and epidermal growth factor receptor (EGFR), on border cells. The lead border cell is thought to receive the highest level of RTK activation ^25^. PVR and EGFR then induce Rac GTPase activity at the front of the cluster ^26^. This in turn leads to accumulation of F-actin in the lead cell and extension of a protrusion ^22, 26^. Ectopic Rac activation in any cell during live migration is sufficient for that cell to produce a stable protrusion and steer the group in a new direction ^26^. Further, the endocytic Rab11 protein promotes cluster-wide cell-cell communication to suppress protrusions in non-leading border cells ^27^. E-cadherin-based tension at the front of the cluster reinforces all of these signals, such that only the front cell forms a protrusion ^28^. The end result is that the border cell cluster moves to the oocyte in a directional manner ^22, 23, 26^.

Finally, it is worth noting some striking similarities and differences between border cell collective migration and a distinctive morphogenetic process that occurs in the developing *Drosophila* eye. The fly eye is made up of repeating units of ommatidia, each of which consists of eight photoreceptor cells surrounded by twelve support cells ^5^. The cells in each ommatidium undergo a collective rotation in which they move together as a group to rotate 90°. The direction of rotation is flipped on opposite sides of the eye imaginal disc midline. Similar to border cell migration, ommatidial rotation is also regulated by RTK signaling, with EGFR being required to complete the full 90° rotation ^68– 70^. However, PCP signaling is the predominant regulator of this polarized collective behavior ^4, 5^, with mutations in core PCP genes such as *fz* causing random rotation of ommatidia ^71^. In border cells, the roles of these two pathways are quite different. Two RTKs, EGFR and PVR, guide border cells to the oocyte and maintain their motility by helping produce front-rear polarity, whereas PCP signaling is less important ^21– 23, 25, 26, 49^.

Both border cell migration and ommatidial rotation also involve E-cadherin as an important effector molecule. The function of E-cadherin in border cells is complex; it promotes migration of border cells upon nurse cells, keeps border cells attached to the central polar cells, and confers cluster-wide polarized tension ^15, 28^. In ommatidia, loss of E-cadherin results in incomplete rotation ^72^. Interestingly, another classical cadherin, N-cadherin, limits rotation, so that ommatidia do not over-rotate. Cadherin regulation ties into the PCP pathway through a MAP kinase family member called Nemo ^72, 73^. Nemo physically binds to the PCP proteins Stbm and Pk, and regulates the activity of E-cadherin complexes during rotation through the cadherin binding partner ß-catenin ^73^. Rho family GTPases, myosin, and diverse F-actin regulatory proteins also function as downstream effectors in the movement of both cell types ^11, 29, 49, 74– 76^.

## Questions for the future

In comparing *Drosophila* border cells and the *Ciona* notochord, several themes emerge that raise further questions. Many developing tissues can simultaneously, or successively, display concurrent types of morphological polarization that include apical-basal, mediolateral, AP, and inside-outside cell polarity. To what extent are these different types of polarity contingent on one another? How often are they truly orthogonal? Dynamic and rapid transitions from one type of cell polarity to another can occur at different stages of development. What are the “switches” between successive phases of cell polarity? And to what extent do they depend on changes in cell-cell communication and gene regulatory networks versus biophysical changes in cell and tissue architecture?

Many tissues exhibit intermediate states of organization that are neither strictly epithelial nor strictly mesenchymal. What is the best way to think about developing tissues that show signs of both epithelial and mesenchymal organization? One likely answer is that intermediate states are important for the ability of cells to move as groups, and to sculpt and remodel organs during embryonic development. Why some developmental processes, but not others, rely on such a mixture of epithelial and mesenchymal properties is currently unclear.

Although the Par/aPKC and PCP pathways are known to organize diverse aspects of polarized cell morphology and behavior, the precise roles and relative importance of these mechanisms vary dramatically in different contexts. Despite an increasingly detailed understanding of the molecular intricacies of the Par/aPKC and PCP pathways, major questions remain as to the mechanisms underlying the temporal and spatial control of the dynamic transitions between successive aspects of cell polarity during development of specific tissues and organs. Dynamic changes in cell and tissue polarity are also central to tumor invasion and metastasis and, while it is well established that polarity proteins and programs are disrupted in many cancer cells ^77, 78^, we generally lack a rich and mechanistic understanding of how this dysregulation occurs.

Answering these and other questions raised by the border cell and notochord systems will help our understanding of how cell polarity contributes to the dynamic formation and remodeling of developing tissues. A better understanding of how cell polarity is established, maintained and transformed will provide a foundation for understanding how stem cells can be used therapeutically to build new organs, and how cancer cells co-opt these programs during tumor progression.
